# Critical Structure Sparing in Stereotactic Ablative Radiotherapy for Central Lung Lesions: Helical Tomotherapy vs. Volumetric Modulated Arc Therapy

**DOI:** 10.1371/journal.pone.0059729

**Published:** 2013-04-05

**Authors:** Alexander Chi, Pan Ma, Guishan Fu, Gerry Hobbs, James S. Welsh, Nam P. Nguyen, Si Young Jang, Jinrong Dai, Jing Jin, Ritsuko Komaki

**Affiliations:** 1 Department of Radiation Oncology, West Virginia University, Morgantown, West Virginia, United States of America; 2 Department of Radiation Oncology, Cancer Hospital and Institute, Peking Union Medical College and Chinese Academy of Medical Sciences, Beijing, China; 3 Department of Biostatistics, West Virginia University, Morgantown, West Virginia, United States of America; 4 NIU Fast Neutron Radiation Therapy Facility, Fermi National Accelerator Laboratory, Batavia, Illinois, United States of America; 5 Department of Radiation Oncology, University of Arizona, Tucson, Arizona, United States of America; 6 Department of Radiation Oncology, The University of Texas MD Anderson Cancer Center, Houston, Texas, United States of America; The University of Chicago, United States of America

## Abstract

**Background:**

Helical tomotherapy (HT) and volumetric modulated arc therapy (VMAT) are both advanced techniques of delivering intensity-modulated radiotherapy (IMRT). Here, we conduct a study to compare HT and partial-arc VMAT in their ability to spare organs at risk (OARs) when stereotactic ablative radiotherapy (SABR) is delivered to treat centrally located early stage non-small-cell lung cancer or lung metastases.

**Methods:**

12 patients with centrally located lung lesions were randomly chosen. HT, 2 & 8 arc (Smart Arc, Pinnacle v9.0) plans were generated to deliver 70 Gy in 10 fractions to the planning target volume (PTV). Target and OAR dose parameters were compared. Each technique’s ability to meet dose constraints was further investigated.

**Results:**

HT and VMAT plans generated essentially equivalent PTV coverage and dose conformality indices, while a trend for improved dose homogeneity by increasing from 2 to 8 arcs was observed with VMAT. Increasing the number of arcs with VMAT also led to some improvement in OAR sparing. After normalizing to OAR dose constraints, HT was found to be superior to 2 or 8-arc VMAT for optimal OAR sparing (meeting all the dose constraints) (*p* = 0.0004). All dose constraints were met in HT plans. Increasing from 2 to 8 arcs could not help achieve optimal OAR sparing for 4 patients. 2/4 of them had 3 immediately adjacent structures.

**Conclusion:**

HT appears to be superior to VMAT in OAR sparing mainly in cases which require conformal dose avoidance of multiple immediately adjacent OARs. For such cases, increasing the number of arcs in VMAT cannot significantly improve OAR sparing.

## Introduction

Stereotactic ablative radiotherapy (SABR), or stereotactic body radiotherapy (SBRT), has been shown to be an excellent treatment option for early-stage non-small cell lung cancer (NSCLC) and lung metastases when a biologically effective dose (BED) of ≥100 Gy_10_ is delivered [1]–[4]. However, treatment related death from severe pulmonary toxicities, hemoptysis, or esophagitis, has been reported when various dose fractionation schedules were delivered to treat centrally located lesions [5]–[9]. This happened mainly when a large fractional dose has been delivered, leading to the overdosing of the organs at risk (OARs) adjacent to the tumor target. Therefore, respecting the OAR dose constraints is essential when treating central lesions close to the mediastinal structures to avoid potentially catastrophic consequences [10].

Excellent OAR sparing has been routinely achieved through intensity modulated radiotherapy (IMRT), which generates highly conformal dose avoidance of structures immediately adjacent to the tumor target in various sites [11], [12]. More recently, advanced techniques of IMRT delivery under image guidance, helical tomotherapy (HT) [13] and volumetric modulated arc therapy (VMAT) [14], [15], have been shown to produce more conformal dose distribution, and better OAR sparing when compared to IMRT, or three dimensional conformal radiotherapy (3D-CRT) at various sites [16]–[24]. Thus, HT & VMAT may be more suitable when treating centrally located lung lesions with SABR.

Previously, we have demonstrated the feasibility of HT-based SABR for centrally located lung lesions which are very close to critical OARs in the thorax; while VMAT has been shown to be superior to IMRT or 3D-CRT for lung SABR in OAR sparing [22]–[25]. In this dosimetric study multi-arc VMAT and HT and compared directly in their ability to maximally spare immediately adjacent OARs when SABR is delivered to the treat centrally located lung lesions. In addition, potential benefits of increasing the number of arcs for VMAT-based SABR in this setting are explored. In this study, 7 Gy × 10 fractions was investigated because it was associated with an excellent toxicity profile when bulky tumors were treated, and the clinically sound BED achieved with this dose fractionation schedule (119 Gy_10_) [26].

## Methods

### Patient and Tumor Characteristics

This study has been approved by the institutional review board (IRB) at the University of Arizona. Since no actual human subjects were involved, no informed consent was needed per IRB. A total of 12 patients with centrally located lesions have been randomly identified. These patients had undergone 3D or intensity-modulated SBRT for stage I-II NSCLC or metastasis to the lung in the department of radiation oncology at the University of Arizona. Central location is defined as the area within 2 cm of the proximal bronchial tree, which includes the lower trachea, carina, mainstem bronchi, and the lobar bronchi. The critical structures are the esophagus, the heart, the spinal cord, major vessels in the mediastinum, and the major airway (lower trachea, carina, mainstem bronchi, and lobar bronchi). The tumor location, size, and its immediately adjacent OARs in each case are listed in Table 1.

**Table 1:** Patient tumor characteristics.

Patients	Location	PTV volume (cc)	Immediately adjacent structures	PTV to structure distance (cm)
1	RLL	153.68	Heart	0.15
2	LUL	70.54	Aortic arch	1.06
			L pulmonary artery	0.11
3	RUL	69.22	Heart	0.50
			SVC	0.23
4	RML	14.04	Heart	1.22
			R middle lobar bronchus	0.10
5	RUL	56.52	R mainstem bronchus	0.20
			R pulmonary artery	0.23
6	RUL	34.91	R brachiocephalic artery	0.19
7	LUL	22.79	Aorta	0.11
8	RUL	133.64	Esophagus	1.30
			SVC	0.26
			Trachea	1.06
9	RUL	147.22	Heart	1.54
			R pulmonary artery	0.39
10	RLL	65.76	Esophagus	0.53
			Heart	0.13
			R pulmonary artery	0.22
11	LUL	24.11	Aortic arch	0.10
12	RML	22.69	Heart	0.23
			R middle lobar bronchus	0.14
			R pulmonary vein	0.97

### Target Volume Delineation

The gross tumor volume (GTV) was delineated at the lung window level on the treatment planning CT. The clinical target volume (CTV) was defined as the GTV and its immediately adjacent areas which were felt to be at a high risk for microscopic disease extension. The planning target volume (PTV) was the CTV with a 5 mm expansion to account for set up errors and residual tumor motion. Particular attention was paid to avoid overlapping any target volumes with the OARs. In cases for which 4D CT was available, internal target motion was accounted for by 4D CT simulation. The lungs, esophagus, spinal cord, and the heart were contoured for each patient. The major vessels and major airway were contoured only when they are adjacent to the GTV. All the target delineation was performed in the clinical Pinnacle treatment planning system, version 9.0 (Philips Medical Systems, Bothell, WA).

### Treatment Planning

Tomotherapy plans were generated in the Tomotherapy Hi-Art planning system using 6 MV photons delivered without a flattening filter. Longitudinal aperture size of 1.05 cm or 2.5 cm, a pitch of 0.3, and a modulation factor of 3 were used. Please refer to our previous study for details [25]. VMAT plans are generated with Smart Arc (SA) using the clinical version 9.0 of Pinnacle to be delivered with 6 MV photons. The machine specification of a Varian linear accelerator with 120 leaf interdigitating MLC is used. VMAT plans were generated with coplanar partial arcs to spare as much contralateral lung as possible. The arc length varied from 150° to 240°. 2-arc and 8-arc plans were created for each case. For 2- arc plans, the delivery time was constrained to 3 minutes. Delivery time was not limited for 8-arc plans. Continuous gantry motion, dose-rate variation, and MLC motion were approximated by optimizing individual beams at 4° gantry angle increments. The machine configuration was based on the “Recommended Smart Arc Physics Parameters” from Philips (Andover, MA. Recommended Smart Arc Physics Parameters. Philips Application Note 2009-03 Rev. A). Except that the “Max MU” limitation, which is 999 by default, has been changed to 5999.

All SABR plans prescribed 70 Gy delivered in 10 daily fractions to the PTV with heterogeneity corrections. They were optimized to have at least 95% of the PTV receiving 100% of the prescription dose with collapsed-cone convolution (CCC) algorithm for both HT and SA. Please refer to our previous study for details on the dose constraints used [27]. PTV coverage took precedence over OAR sparing in all plans. All treatment plans were designed under the same set of planning guidelines agreed upon among the authors with similar levels of emphasis placed on the PTV and the OARs. HT planning was conducted at the University of Arizona, and VMAT planning was conducted at the Cancer Hospital & Institute at Peking Union Medical College. In addition, all the plans were designed to deliver a dose that is used in daily clinical practice.

### Plan Comparison

**Figure 1:**
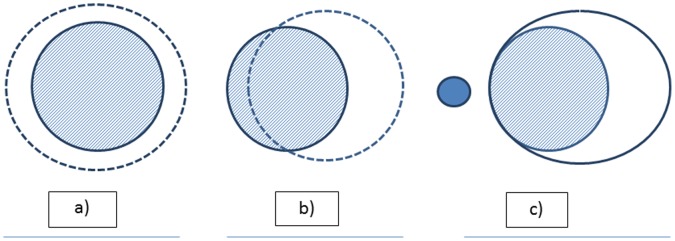
Illustration of the possible scenarios of dose conformity described by the conformation number (CN). The shade represents the target volume, the dotted line represents the desired isodose, the small solid in c) represent a critical structure that is immediately adjacent to the target. a). the ideal dose conformation with CN = 1. b). Less than optimal coverage of the target volume. c). In situations where the target is next to a critical structure, both adequate dose coverage of the target and the sparing of the critical structure are desired. As a result, more healthy tissue is irradiated in the context of the healthy tissue dose constraint as shown. The CN will be <1 is both b) and c).

Various lung dose parameters and the maximum dose (D_max_) to specific OARs generated in HT and VMAT plans were compared to assess their ability for OAR sparing. For the PTV, the dose covering 95% of the PTV (D_95_), the % PTV receiving ≥70 Gy (V_70 Gy_), the mean dose (D_mean_) & D_max_, the homogeneity index (HI), and the conformation number (CN) were generated and compared between different techniques. The HI and CN are previously defined [28], and are described below:
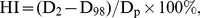
(1)

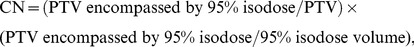
(2)D_2_ and D_98_ represent the doses to 2% and 98% of the PTV, D_p_ is the prescription dose. For the CN, the first portion (1^st^ parentheses) is an assessment of target volume coverage by 95% of the prescription dose; and the second part (2^nd^ parentheses) is an assessment of normal tissue sparing (the volume of normal tissue receiving ≥95% of the prescribed dose). The CN values between 0 and 1 with 1 representing the ideal conformity (Fig. 1a). In other situations that may be encountered in SABR delivery, a less-than-ideal CN is achieved when the target is partially covered by the desired dose with proportionately increased irradiation of the healthy tissue (Fig. 1b); or increased volume of healthy tissue may be irradiated within the limit of the allowed dose constraints due to the need to adequately cover the target volume and to spare a critical structure that is in its proximity (Fig. 1c).

The differences in tumor characteristics, such as tumor size and the distance between the PTV & its immediately adjacent structures, were sought between the group of patients for whom optimal PTV coverage and OAR sparing was achieved (Group 1) and those whose plans were suboptimal (Group 2). Group 1 included both HT and VMAT plans (2 and/or 8 arc plans). Group 2 included patients for whom either the HT or both VMAT plans could not successfully spare ≥1 immediately adjacent OAR if adequate PTV coverage was maintained.

### Statistical Analysis

Dosimetric parameters generated in the HT, 2-arc, and 8-arc plans were compared through a randomized complete block ANOVA. After the dose parameters for the OARs and the target volumes were obtained from the HT and the VMAT plans, they were normalized to the OAR dose constraints listed in Table 2. Selected OAR dose parameters from the HT, and VMAT plans were compared using multifactorial ANOVA while controlling for differences between patients and various OARs. In the assessment of each treatment technique’s influence on OAR sparing, multiple logistic regression was then performed on these selected normalized parameters. In analyzing the differences in tumor characteristics between groups 1 & 2, one-way ANOVA was used. Statistical significance was defined by a *p* value <0.05. All analyses were performed using JMP-Pro/v9.0.2 (SAS Institute Inc., Cary, NC).

**Table 2:** Comparison of PTV dose coverage parameters generated through helical tomotherapy, VMAT with 2 arcs, and 8 arcs with absolute doses illustrated in mean ± standard deviation.

			VMAT			*P* value	
		HT	2 Arcs	8 Arcs	HT vs. 2 Arcs	HT vs. 8 Arcs	2 Arcs vs. 8 Arcs
*PTV dose coverage parameters*							
D_95_ (Gy)	70.61±0.61		70.00±0.00	70.00±0.00	0.0003	0.0003	0.99
V_70 Gy_ (%)	96.15±1.22		95.00±0.00	95.00±0.00	0.0006	0.0006	0.99
D_mean_ (Gy)	74.03±1.74		76.12±1.53	74.99±1.69	0.002	0.11	0.06
D_max_ (Gy)	81.98±3.66		82.76±2.77	80.76±2.39	0.46	0.26	0.07
CN	0.64±0.06		0.61±0.11	0.63±0.11	0.17	0.73	0.29
HI	19.23±10.95		21.45±6.66	17.83±5.75	0.24	0.45	0.06

## Results

HT and VMAT SABR plans were generated for all 12 patients to meet the PTV dose coverage criteria. The PTV, lung, and other OARs’ dose parameters generated with each treatment approach are summarized in Tables 2, 3, and -4, respectively, with any two different techniques compared directly.

**Table 3:** Comparison of lung dosimetric parameters generated through helical tomotherapy, VMAT with 2 arcs, and 8 arcs with absolute doses illustrated in mean ± standard deviation.

			VMAT			*P* value	
		HT	2 Arcs	8 Arcs	HT vs. 2 Arcs	HT vs. 8 Arcs	2 Arcs vs. 8 Arcs
*Total lung*							
MLD (Gy)	6.48±2.33		7.23±3.47	6.50±2.53	0.24	0.97	0.25
V_5_	21.16±8.03		22.89±10.15	22.31±9.25	0.23	0.42	0.68
V_10_	15.88±5.48		15.16±6.18	14.62±5.78	0.38	0.13	0.51
V_20_	10.49±4.16		10.27±4.12	9.94±3.86	0.82	0.57	0.73
*Ipsilateral lung*							
MLD (Gy)	10.72±3.67		12.70±4.98	12.03±4.50	0.03	0.15	0.45
V_5_	34.10±13.29		38.22±14.09	37.64±13.66	0.03	0.06	0.75
V_10_	28.35±11.11		30.19±10.90	27.93±10.36	0.42	0.15	0.32
V_20_	19.50±8.81		21.42±8.06	20.78±7.68	0.36	0.54	0.76
*Contralateral lung*							
MLD (Gy)	1.62±0.75		1.64±0.76	1.60±0.67	0.90	0.95	0.85
V_5_	7.04±6.27		8.76±7.10	8.10±5.70	0.36	0.57	0.72
V_10_	2.11±2.88		1.11±1.11	0.98±1.16	0.06	0.04	0.79
V_20_	0.27±0.42		0.04±0.09	0.05±0.07	0.04	0.04	0.98

### HT vs. 2-arc VMAT

For the PTV, D_95_ and V_70 Gy_ were significantly higher in the HT plans (Table 2). HT also generated significantly lower D_mean_ (*p* = 0.002). No significant difference in the D_max_, CN, and HI was found. For the total lung (volume of both lungs – GTV), no significant differences in the mean lung dose (MLD), V_5_, V_10_, and V_20_ was observed (Table 3). The ipsilateral MLD and V_5_ were significantly lower in HT plans (*p* = 0.03, 0.03, respectively). On the contrary, 2-arc VMAT achieved lower V_20_ in the contralateral lung (*p* = 0.04). HT generated significantly lower D_max_ for the heart and the major vessels (*p* = 0.02, 0.01, respectively), while a trend toward lower Dmax for the major airway was observed (*p* = 0.07) (Table 4). However, lower D_max_ to the spinal cord was found in VMAT plans (*p* = 0.03).

**Table 4:** Comparison of the maximum dose other organs at risk (OARs) generated through helical tomotherapy, VMAT with 2 arcs, and 8 arcs with absolute doses illustrated in mean ± standard deviation.

			VMAT			*P* value	
		HT	2 Arcs	8 Arcs	HT vs. 2 Arcs	HT vs. 8 Arcs	2 Arcs vs. 8 Arcs
*D_max_ for other OARs (Gy)*							
Spinal cord	18.84±7.44		14.18±8.57	13.65±8.20	0.03	0.02	0.79
Esophagus	22.72±13.06		22.71±13.52	22.28±11.84	0.99	0.62	0.62
Heart	23.08±22.83		29.77±26.70	29.66±26.54	0.02	0.03	0.97
Major airway	34.10±15.02		37.97±17.37	37.30±17.09	0.07	0.12	0.74
Major vessels	46.30±3.20		50.91±6.51	49.03±5.35	0.01	0.09	0.24

### HT vs. 8-arc VMAT

Significantly higher D_95_ and V_70 Gy_ for the PTV for HT was the only observed difference in the target dose indices (Table 2). Total-lung dose parameters were equivalent, while the contralateral V_10_ & V_20_ was significantly lower in VMAT plans (*p* = 0.04, 0.04, respectively) (Table 3). For other OARs, HT achieved lower D_max_ to the heart, while VMAT achieved lower D_max_ to the spinal cord (*p* = 0.03, 0.02, respectively) (Table 4).

### 2-arc vs. 8-arc VMAT

For the PTV, a trend toward significance for lower D_mean_, D_max_, and HI were observed (*p* = 0.06, 0.07, 0.06, respectively) (Table 2). No significant difference between the 2 & 8-arc plans was found in any of the OAR parameters (Tables 3 & 4).

### D_max_ for Adjacent OARs and MLD, V20 for the Total Lung ((MLD_total_, V_20, total_) after Normalizing to the Dose Constraints Used

**Figure 2:**
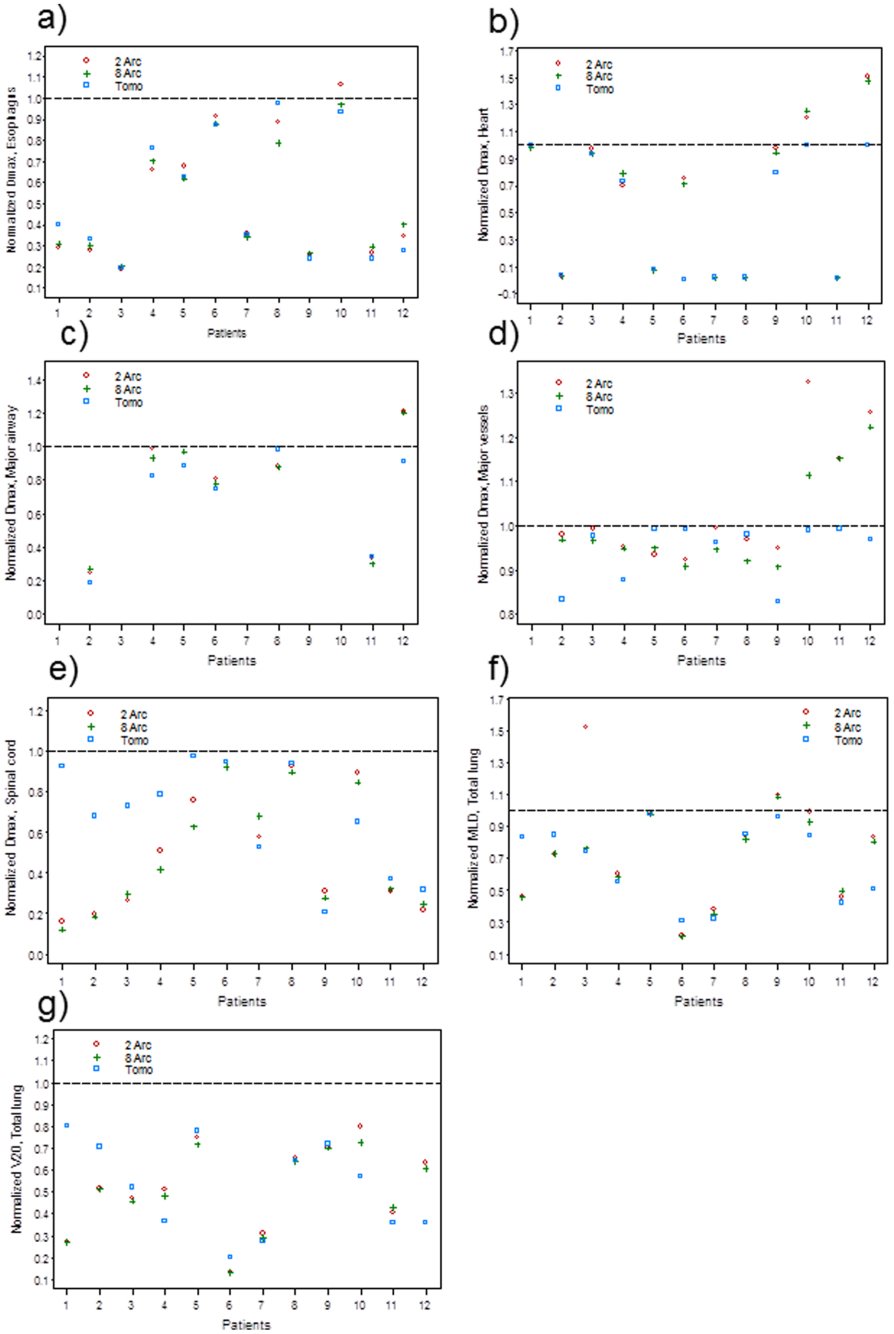
Comparison of dose parameters to the organs at risk. Organs at risk: a) Esophagus, b) heart, c) major airway, d) major vessels, e) spinal cord, f) and g) mean lung dose (MLD) and V_20_ for the total lung, after normalized to the absolute dose constraints between helical tomotherapy (Tomo), 2-arc, and 8-arc VMAT plans.

No statistically significant difference between the three different techniques was observed in the normalized dose parameters when patient and OAR differences were controlled (Fig. 2). However, the treatment technique was found to be a statistically significant factor influencing OAR sparing (meeting dose constraints), favoring HT for all OARs as an aggregate, when compared with VMAT techniques (*p* = 0.0004); specifically affecting MLD_total_ (*p* = 0.0219), and D_max_ to the heart (*p* = 0.0219) & the major vessels (*p* = 0.0033). OAR sparing was successfully achieved in all HT plans. However, OAR overdosing was found in 2 &/or 8-arc plans in patients 3, 9, 10, 11, 12 (Fig. 2). Increasing from 2 to 8 arcs decreased the esophageal D_max_ for patient 10, and the MLD_total_ for patient 3 to below the dose threshold (Fig. 2a, f). The same did not occur for the heart, the major airway, the major vessels, and the MLD_total_ for patients 9, 10, 11, and 12 (Fig. 2b, c, d, and f). However, increasing from 2 to 8 arcs decreased the D_max_ to the major vessels and the spinal cord in many cases (Fig. 2d, e).

### Tumor Characteristics for Patients for Whom Neither VMAT Plans Achieved Optimal OAR Sparing

The tumor characteristics for group 2 (patients 9–12) were compared with those for group 1 (patients 1–8). Given the small sample size for each group, no significant difference was found in PTV diameter, volume, and distances to the closest and furthest immediately adjacent OARs (Table 5). 2/4 cases in group 2 had 3 immediately adjacent structures (50%), while only 2 such cases were found in group 1 (25%). The median PTV to its closest OAR distance was also slightly shorter in group 2 when compared with group 1 (0.13 cm vs. 0.17 cm).

**Table 5:** Tumor characteristics for groups of patients for whom optimal OAR sparing and tumor volume dose coverage can be achieved with HT or any one form of VMAT (Group 1) and those patients among whom optimal OAR sparing cannot be achieved if optimal tumor volume coverage is desired with VMAT (Group 2).

	PTV diameter (cm)	PTV volume (cc)	Number of immediately adjacent normal structures	Shortest distanceto the PTV (cm)	Longest distance to thePTV (cm)
Group 1	6.05±1.90	69.42±50.46	1.88±0.84	0.17±0.06	0.61±0.50
Group 2	5.94±1.74	65.07±58.61	2.25±0.96	0.19±0.13	0.79±0.62

## Discussion

**Figure 3:**
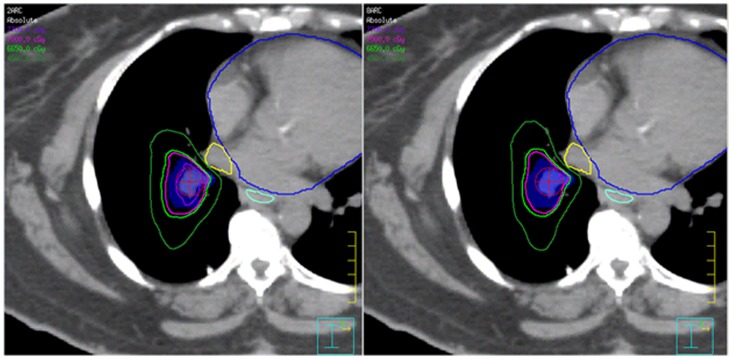
Illustration of a comparison of the 2 and 8 Arc plans demonstrating that the shape of the isodose covering the PTV is largely dependent on the immediately adjacent critical structures (yellow and blue) that need to be spared in one patient. As a result, slightly increased volume of the normal lung tissue is included in the high dose volume lateral to the PTV (blue shade) away from the central structures. Also shown here is that when comparing to the 2 Arc plan, the high dose region included by the 77 Gy isodose in the 8 Arc plan is greatly diminished, demonstrating increased homogeneity.

Although higher D_95_ and V_70 Gy_ were found in HT plans, this is mostly due to the differences in how target coverage parameters were executed in the treatment planning systems (TPS) under comparison. No significant difference in dose conformality was found between HT and VMAT plans. However, increasing from 2 to 8 arcs led to a trend toward lower PTV D_mean_, D_max_, and HI (Table 2). Thus, suggesting a potential for improving dose homogeneity by increasing the number of arcs when treating targets in areas of complex geometry with VMAT. This finding is consistent with what has been previously observed by Guckenberger et al [29]. Poor CN has been found with all three different techniques (Table 2). The CN achieved in our VMAT plans was lower than what has been reported in the literature [22]. This may be partially due to the degree of complexity in OAR sparing in close vicinity to the target; which is especially true when multiple OARs are immediately adjacent to the PTV, making it extremely difficult to conform the dose to the PTV in all directions. In these cases, less conformity is observed due to increased dose to the healthy tissue that has the least demanding dose constraint in the context of each specific case as previously shown in Fig. 1c. This is also illustrated in Figure 3, where the desired isodose can be seen to include additional normal lung tissue due to the sparing of immediately adjacent normal structures. In the same illustration, increased dose homogeneity from the 8-Arc plan is also shown as the 77 Gy isodose is significantly diminished when compared with the 2-Arc plan. Thus, supporting that dose homogeneity may be improved by increasing the number of arcs.

Lung dose parameters in HT and VMAT plans are shown in Table 3. VMAT plans demonstrated significantly lower contralateral lung dose parameters when compared to HT plans (Table 3). This can possibly be explained by the difference in the degree of the arc generated in 2 different TPS, which is partial arc for VMAT and full arc for HT. Due to the already very low values of the contralateral lung dose parameters, VMAT’s potentially improved contralateral lung sparing may not be of any clinical significance. For the ipsilateral lung, potential factors of clinical relevance, the V_5_, and MLD, were significantly lower in HT plans when compared to 2-arc plans [30]. However, this significance was lost when HT and 8-arc plans were compared. Similar to what was observed for the ipsilateral lung, HT has demonstrated significantly lower D_max_ to the major vessels than 2-arc VMAT. However, this significance was lost when HT was compared to 8-arc VMAT. Although no significant difference was observed in any OAR dose parameters between 2 & 8 arcs VMAT plans, these observations again suggest that increasing from 2 to 8 arcs for VMAT-based SABR may have a potential for improving conformal dose avoidance in areas of complex geometry.

With patient and OAR differences controlled, no difference in a series of normalized dosimetric parameters was found between the three techniques (Fig. 2). However, the technique used was found to be a significant factor influencing the ability to meet OAR dose constraints favoring HT over VMAT. Optimal OAR sparing was achieved in all cases by HT, but only the first 8 cases for VMAT (Fig. 2). Increasing from 2 to 8 arcs helped to meet the dose constraints for certain structures for patients 3 & 10 (Fig. 2 a, f), and decreased D_max_ for the spinal cord and the major vessels in many cases (Fig. 2 d, e). This did not lead to meeting the dose constraints for all immediately adjacent OARs for patients 9–12, among whom increased number of immediately adjacent structures and short distance between PTV and the closest OAR were common. No statistically significant difference in tumor characteristics was found between patients 9–12 and patients 1–8 due to the small number of patients studied (Table 5). However, our findings suggest that HT may be more appropriate in cases which demand conformal dose avoidance of multiple structures (≥2) that are very close to the PTV in the delivery of SABR for centrally located lung lesions, even though increasing the number of arcs in VMAT may improve OAR sparing in certain situations. Our findings are corroborated in a study comparing HT and VMAT in delivering conventionally fractionated radiotherapy in several body sites by Rong et al, which demonstrated improved target dose homogeneity and lower doses to more critical structures in the HT plans [28]. This is mainly due to the increased freedom of intensity modulation created by delivering image-guided IMRT under synchronous gantry rotation and couch motion with HT [13], [25].

Although shown to be more capable of OAR sparing in setting of lung SABR for central lesions, HT is associated with much longer fractional treatment delivery time of >40 minutes for each case, mainly attributing to the complexity of intensity modulation required. Due to this fact, the exact treatment time for the HT plans was not recorded. On the other hand, 2 & 8 arc VMAT had average fractional treatment delivery times of only 180 & 331 seconds, respectively. Thus, VMAT remains to be more desirable for targets in areas of relatively less complex geometry. The prolonged treatment time associated with HT can be potentially improved by implementing the dynamic jaw and dynamic couch feature [31]. At the current time, this remains a problem for HT-based SABR mainly because of its associated increase in intrafractional motion, which can be critical when treating central lesions with SABR. As a result, proper respiratory motion management and careful body immobilization are essential [32], [33]. In our experience, 4D CT simulation to account for tumor motion in various locations remains the most straight forward approach for respiratory motion management for HT clinically. Furthermore, treatment efficiency can be improved by dividing the fractional dose into two consecutive fractions (7 Gy delivered in 2 consecutive fractions, 3.5 Gy/fraction) [34].

The dose delivery & calculation accuracy have been commented elsewhere, which were found to be adequate for both VMAT and HT [28]. In a study by Takahashi et al, the CCC algorithm closely approximated the Monte Carlo algorithm in the dose calculation specific for lung SBRT [35]. This warrants the validity of SABR dose calculation for both HT and VMAT, which is also critical in the setting of centrally located lesions closely surrounded by multiple critical OARs. Early clinical reports on treating mostly peripheral lesions with HT-based and VMAT-based SABR have been promising [34], [36]. A prospective clinical study investigating how to best apply these advanced techniques in the treatment of central lung lesions with SABR will be conducted in the near future.

Due the virtual nature of this dosimetric study, no further quality assurance is conducted. However, treatment planning accuracy for VMAT and HT are implied from studies conducted in the past (25, 37). But it will be done as part of a prospective study in the future.

### Conclusion

In delivering SABR or SBRT for centrally located lung lesions, HT appears to be superior to VMAT in OAR sparing mainly for targets with multiple immediately adjacent structures. Although increasing arc number cannot achieve the aim of sparing all the OARS in cases associated with complex geometry, it may help lowering the doses to them. However, VMAT may be preferred over HT in cases of simpler geometry due to much shorter treatment delivery time.
